# Unsplinted Attachments and Patient-Reported Outcome Measures (PROMs) in 2-Implant-Retained Mandibular Overdentures: A Systematic Review

**DOI:** 10.1155/2022/5955847

**Published:** 2022-05-24

**Authors:** Pravinkumar G. Patil, Liang Lin Seow, Ting Jing Kweh, Smita Nimbalkar

**Affiliations:** ^1^Division of Restorative Dentistry, School of Dentistry, International Medical University, Kuala Lumpur, Malaysia; ^2^Division of Clinical Oral Health Sciences, School of Dentistry, International Medical University, Kuala Lumpur, Malaysia

## Abstract

**Introduction:**

The purpose of this review is to compare randomized clinical trials evaluating the patient-reported outcome measures (PROMs) using different unsplinted attachment systems in 2-implant-retained mandibular overdentures (2IRMODs). A focus question (as per PICOS) was set as follows: does one particular unsplinted attachment system (I) compared with another (C) result in better patient-reported outcomes (O) in two-implant-retained mandibular overdentures (P) using randomized controlled trials (S)?

**Materials and Methods:**

A literature search was conducted in the PubMed MEDLINE and Cochrane Central Register of Controlled Trials (CENTRAL) databases between November 2010 and October 2020. Only randomized controlled trials (RCTs) on 2IRMOD using unsplinted attachment systems measuring patient-centered outcomes were selected. A total of 171 studies were identified in initial search, and 27 studies were shortlisted for full-text evaluation. A total of 5 studies were included for a systematic review. The risk of bias was evaluated using Cochrane Risk of Bias Tool 2.0 (RoB 2.0). Meta-analysis could not be performed as different studies evaluated different patient-reported outcomes, namely, satisfaction, quality of life, complications, preferences, or combinations of these.

**Results:**

A total of 23 patients received low-profile (self-aligning) attachments (in 2 studies), 69 patients received standard ball attachments (in 5 studies), 25 patients received telescopic (or conus) attachments (in 2 studies), and 20 patients received mini-ball attachments (in 1 study). Two studies compared ball attachments and low-profile attachments and revealed similar satisfaction and quality of life (QoL). Two studies compared ball attachments with telescopic attachments and revealed less patient satisfaction in telescopic attachments. A single study compared mini-ball attachments with standard ball attachments and showed no difference in patient-reported outcomes. Three studies were found to have a low risk of bias, and the remaining two studies had a high risk of bias.

**Conclusions:**

The standard ball, mini-ball, and low-profile attachments have no influence on PROMs in the normal interarch space. Inconclusive results were found in studies that evaluated PROMs using ball attachments versus telescopic attachments.

## 1. Introduction

### 1.1. Background

Completely edentulous patients experience an impaired ability to perform essential life tasks, such as speaking and eating [1, 2]. Dental implants have provided varieties of fixed abutments and/or removable attachment systems in restoring completely edentulous arches in recent years to overcome the problem of retention and stability of conventional complete dentures [3–5]. Clinically, a stable mandibular denture was the most important determinant of patients' satisfaction [6]. During the symposium at McGill University, Canada (in 2002), an expert consensus statement [7] was released that states ‘‘There is now overwhelming evidence that a two-implant overdenture should become the first choice of treatment for the edentulous mandible.” This consensus was based on clinical evidence reporting a significant improvement in the quality of life (QoL) of the two-implant-retained mandibular overdentures (2IRMODs) against conventional complete denture users. The statement was later supported by the experts in the England symposium (in 2009) and the US academic prosthodontic experts' opinions survey (done in 2011) [8].

### 1.2. Types of Overdenture Attachments

Attachment systems are an integral part of the implant overdentures and broadly classified into 4 categories, namely, bar, stud, magnetic, or telescopic [9, 10]. Three attachment types (stud, magnetic, or telescopic) were considered as unsplinted or free-standing attachments, and the bar was considered as a splinted attachment. The bar attachment systems are made up of metallic casted or milled bars (usually in a semicircular cross section) joining two or more implants providing the splinting effect to the implants. In patients with a decreased vertical dimension or reduced vertical restorative space, the free-standing or unsplinted attachment systems are used over the splinted (bar-clip) type and are beneficial in terms of initial treatment cost, hygiene, and simplicity in the manufacturing process [11, 12]. In recent years, the different stud attachment designs have been introduced, namely, Locator by Zest Anchors, Equator by Rhein83, and ERA by Sterngold. These newer attachments are known by their trade names; however, the generic category is still considered as the stud attachments [12–14]. All these attachments can accommodate limited interarch space as low as 2.5 mm (claimed by the manufacturer/s) and hence are also referred to as low-profile attachments [12]. These attachments can also be used in nonparallel implant angulations (<30°) and hence are called as self-aligning attachments.

### 1.3. What Is Already Known and What Is the Need for This Review

Miler et al. [15] have carried out a systematic review of 10 clinical studies involving low-profile attachment (Locators) and concluded that Locators provided acceptable patient satisfaction. Keshk et al. [16] systematically reviewed 3 randomized clinical trials (RCTs) on telescopic attachments and ball attachments and observed no significant difference in prosthodontic maintenance. Gonçalves et al. [17] performed a systematic review and evaluated 16 randomized clinical trials comparing either bar and clip or ball and O-ring attachments and concluded that all the three have similar clinical performance regarding mechanical and functional properties and patient satisfaction. Even though these newer low-profile stud attachments were in dental practice for almost 2 decades, these attachments were not being compared enough against their conventional counterpart of ball attachments. To the best of the authors' knowledge, there is no systematic review carried out exclusively to compare patient-reported outcome measures (PROMs) with different unsplinted attachment systems in the 2IRMOD.

### 1.4. Focus Question (PICOS) and Objectives

A focus question (as per PICOS) was set as follows: Does one particular unsplinted attachment system (I) compared with another (C) result in better patient-reported outcomes (O) in two-implant-retained mandibular overdentures (P) using randomized controlled trials (S)? The objective of this systematic review was to determine the patient-reported outcomes with different unsplinted attachment systems for 2IRMOD.

## 2. Materials and Methods

### 2.1. Review Registry and Ethical Approval

This systematic review evaluated the randomized clinical trials comparing different unsplinted attachment systems used in 2IRMOD. The study was registered in the Prospective Register of Systematic Reviews (PROSPERO) platform (CRD42020180606). The study was conducted according to the Preferred Reporting Items for Systematic Reviews and Meta-Analyses (PRISMA) checklist. Institutional ethical approval has been obtained from the authors' institute (Project ID: 497/2020).

### 2.2. Eligibility of Studies

The following inclusion criteria were applied to select the studies: (1) Participants: completely edentulous patients treated with 2IRMOD. (2) Intervention: 2IRMOD with unsplinted attachments (without considering implant type, manufacturer, and surgical or prosthetic protocols). (3) Comparison: studies comparing different unsplinted attachments against each other. (4) Outcome: patient-centered outcomes including quality of life (Qi), satisfaction, masticatory performance, and complications. (5) Study types: randomized clinical trial (RCT) studies. The studies where the single type of attachments was compared or the group was compared with splinted attachment systems were excluded.

### 2.3. Search Strategy

The electronic literature search was conducted independently by 2 researchers (PGP, KTJ) in the Cochrane Central Register of Controlled Trials (CENTRAL) and PubMed MEDLINE between 1^st^ November 2010 and 31^st^ October 2020 (Table 1). A literature search was also performed in ClinicalTrials.gov and the WHO International Clinical Trials Registry. A manual search was also performed which did not reveal any additional eligible study.

### 2.4. Risk of Bias

The selected studies were appraised by two reviewers (TJK, SN) independently in the 5 domains, namely, the randomization process, deviations from intended interventions, missing outcome data, measurement of the outcome, and selection of the reported result using revised Cochrane Risk of Bias Tool 2.0 (RoB 2.0). Any disagreement was resolved after discussion with the third reviewer (SLL). Individual studies were categorized as high or low risk of bias or some concerns. For clinical trials that evaluated the same study population, only the study with the higher observation time was included.

### 2.5. Summary of Studies

The data were extracted on variables such as study method, participants, intervention, and outcome by two reviewers (TJK, PGP) and combined for analysis. The summary of selected information was tabulated based on predetermined criteria to facilitate the effect of attachment systems. Meta-analysis could not be performed as different studies evaluated different patient-reported outcomes namely satisfaction quality of life, complications, preferences, or combinations of these. The level of agreement between the reviewers regarding relevant factors in the studies was determined using Cohen's kappa coefficient (*κ*).

## 3. Results

### 3.1. Study Selection

**Figure 1:**
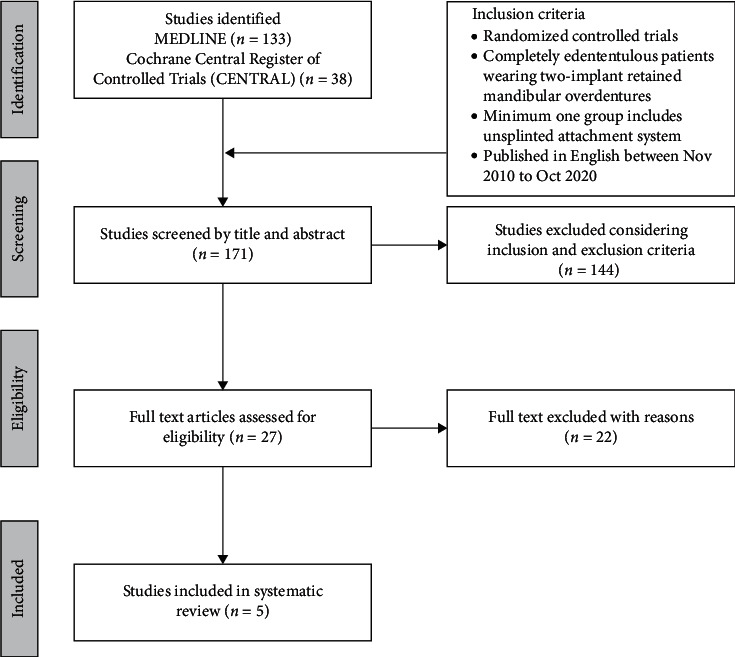
Study selection process (PRISMA) checklist.

A total of 133 studies were identified in the initial search, and 27 studies were shortlisted for full-text evaluation (Figure 1). After full-text evaluation, a total of 22 studies [18–39] were excluded due to different reasons (Table 2). These studies [18–39] were excluded mainly because of either unsplinted attachments were compared directly with splinted attachments or only one type of unsplinted attachment was used in different groups or the clinical parameters were not related to the attachments (Table 2). For the study selection process, the kappa coefficient value (*κ* = .83) indicated a high level of agreement between the 2 reviewers (TJK and SN).

### 3.2. Summary and Characteristics of the Studies

Final 5 studies [40–44] were included for a systematic review, and the findings are summarized in Table 3. A total of 23 patients received low-profile (self-aligning) attachments (in 2 studies) [40, 41], 69 patients received standard ball attachments (in 5 studies) [40–44], 25 patients received telescopic or conus attachments (in 2 studies) [42, 43], and 20 patients received mini-ball attachments (in 1 study) [44]. Two studies [40, 41] were crossover randomized trials where a period of 3 months was given to use either ball or low-profile attachment and another period of 3 months for alternate attachments. Three [42–44] studies were randomized clinical trials with the follow-up period of 5 years [42], 3 years [43], and 6 months [44]. The standard-sized implants were used in all groups with different manufacturers in four studies [40–43], and the mini-implants were used as one of the groups in a single study [44]. Early or delayed prosthetic loading protocols were observed within 1 and a half month [40], 3 months [41–43],^,^ and 2 months [44]. One study [44] evaluated both QoL and patient satisfaction, three studies [41–43] evaluated only patient satisfaction, and a single study [40] evaluated only QoL.

### 3.3. Effect of Attachments on Different Types of PROMs

Two studies [41, 42] evaluated the postinsertion maintenance, of which a single study [41] observed no difference between ball attachments and low-profile attachments and another study [42] observed significantly higher need of the matrix repairs and activation. All studies found out comparable performances against each other regarding either patient satisfaction, prosthetic maintenance, or overall clinical performance. All the studies included in the present review have a standard ball attachment group as a constant comparator against either low-profile, telescopic, or mini-ball attachments [40–45]. All studies compared PROMs with different implant systems, loading protocols, and recall periods. All studies, except one study, have observed similar PROMs when compared amongst ball, telescopic, low-profile, and mini-ball attachments. A single study observed high dissatisfaction with the telescopic (conus) attachment and resulted in numerous patients refusing to further participate in the study. Except for the deceased participants, all patients stayed with the ball attachment system, whereas only 7 of 11 patients stayed with the conus system. This interpretation can be used cautiously because a limited number of patients were studied. The single study [40] observed that low-profile (self-aligning) attachments were comparable to ball attachments in oral health-related QoL in the normal interarch space but may be superior in cases of reduced space for attachment placement.

### 3.4. Risk of Bias

**Figure 2:**
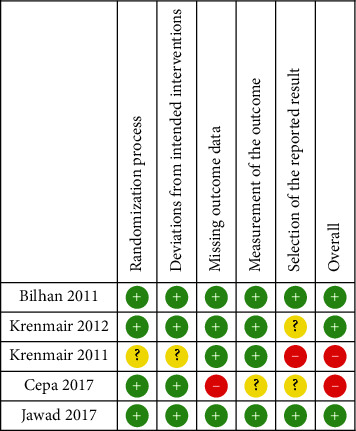
Risk of bias of each selected study.

**Figure 3:**
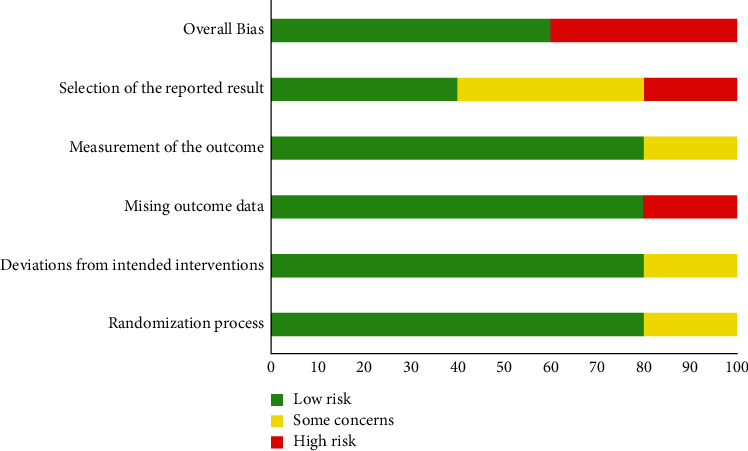
Overall type of risk of bias of included studies.

The final risk of bias assessment of the included studies is illustrated in Figures 2 and 3. Two studies [42, 43] were judged to have a high risk of bias, and three [40, 41, 44] were judged to a have low risk of bias based on the RoB 2.0 analysis (Figure.  2, 3).

## 4. Discussion

### 4.1. Ball Attachment as a Common Group

This systematic review provided understanding amongst the RCTs carried out with direct comparison between 2 different unsplinted attachment systems studied with any pair of combination. All 5 studies evaluating PROMs have a common group of a standard ball attachment [40–44] which was compared against either low-profile [40, 41], telescopic [42, 43], or mini-ball [44] attachments in 2IRMOD. Regarding PROMs, a limited number of studies have indicated different results with different unsplinted attachment pairs of comparison (ball versus low-profile or telescopic or mini-ball) under different study conditions (implant manufacturers, loading protocols, and follow-up periods), leading to inconclusive remarks. Despite many confounding factors and the limited number of studies, few concluding remarks can be drawn in relation to PROMs which could be translated in the clinical practice. The impact of implant-retained overdentures on QoL is identified as an essential outcome [45]. Oral health-related QoL is a comprehensive and multifactorial evaluation of oral diseases. The Oral Health Impact Profile (OHIP) is one of the most valid and reliable tools used to evaluate the oral health-related QoL [46].

### 4.2. Confounding Factors in Measurement of PROMs in 2IRMOD

Several confounding factors could possibly influence the overall outcomes of the unsplinted attachments. These factors are implant manufacturers, loading protocols, types of PROMs (namely, patient satisfaction, QoL, preferences, masticatory performance, reporting of the complications, denture stability/retention, or maintenance), method of evaluation, language, and patient demographics.

### 4.3. The Limitations and Future Directions

This review included only 5 studies due to a limited number of clinical trials published. Two studies have indicated a high risk of bias. Hence, the results of this systematic review should be interpreted cautiously. More number of studies suggested reporting of PROMs with implant overdentures using unsplinted attachments, especially masticatory performance, oral health-related QoL, and prosthetic complications. The low-profile attachments (Locator (Zest Anchors), Equator (Rhein83), and ERA (Sterngold)) [12–14] have ability to accommodate limited interarch space and can take up interimplant angulations as much as 30° [12]. Miler et al. [15] reviewed 10 clinical studies involving the low-profile attachment (Locator); however, not many clinical trials are carried out comparing other low-profile attachments. More clinical trials are recommended for comparing magnets and telescopic attachments as well as low-profile attachments such as Equator and ERA. The parameters included in different types of PROMs and related evaluation tools used are not uniform. The standard guidelines should be developed in regard to the tools used in evaluating and reporting different types of PROMs.

## 5. Conclusions

Within the limitations of this systematic review, the following conclusions were drawn. The standard ball, mini-ball, and low-profile attachments have no influence on PROMs in normal interarch space. In reduced interarch space, low-profile attachments demonstrated better PROMs. Inconclusive results were found among the studies that evaluated PROMs in patients using ball attachments versus telescopic attachments.

## Figures and Tables

**Table 1:** Search strategy.

Database	Search strategy
PubMed MEDLINE (*n* = 133)	(((((((((((“denture, overlay” [MeSH Terms] OR (“denture” [All Fields] AND “overlay” [All Fields]) OR “overlay denture” [All Fields] OR (“denture” [All Fields] AND “overlay” [All Fields]) OR “denture overlay” [All Fields]) AND “dental prosthesis, implant-supported” [MeSH Terms]) OR “dental implants” [MeSH Terms] OR “dental implant abutment design” [MeSH Terms]) AND “jaw, edentulous” [MeSH Terms]) OR “mouth, edentulous” [MeSH Terms]) AND “mandible” [MeSH Terms] ) AND (locator)) OR (equator)) OR (conus)) OR (unsplinted attachment)) OR (ball attachment)) OR (telescopic crown) Filters applied: Clinical Trial, Randomized Controlled Trial, from 2010/11/1 - 2020/10/31

Cochrane Central Register of Controlled Trials (CENTRAL) (*n* = 38)	#1 MeSH descriptor: [Jaw, Edentulous] explode all trees 603 #2 MeSH descriptor: [Mouth, Edentulous] explode all trees 773 #3 MeSH descriptor: [Mandible] explode all trees 1653 #4 MeSH descriptor: [Denture, Overlay] explode all trees 344 #5 MeSH descriptor: [Dental Prosthesis, Implant-Supported] explode all trees 789 #6 #1 OR #2 OR #3 OR #4 OR #5 2458 #7 MeSH descriptor: [Denture Precision Attachment] explode all trees 31 #8 (ball attachment):ti,ab,kw OR (locator):ti,ab,kw OR (magnet):ti,ab,kw OR (equator):ti,ab,kw OR (telescopic):ti,ab,kw 992 #9 (unsplinted attachment):ti,ab,kw OR (nonsplinted attachment):ti,ab,kw OR (overdenture attachment):ti,ab,kw 108 #10 #7 OR #8 OR #9 1032 #11 (“patient-reported outcome measures”) 1697 #12 (Quality of life) 129196 #13 (patient satisfaction) 39727 #14 (masticatory performance) 141 #15 #11 OR #12 # OR 13 OR #14 337514 #16 #6 AND #10 AND #15 38

**Table 2:** Excluded studies with reasons.

Sr. no.	Authors	Year	Reason for exclusion
1	Patil et al. [18]	2020	Only Locator attachments compared
2	Burns et al. [19]	2011	Compared directly with splinted attachments
3	Zygogiannis et al. [20]	2018	Compared directly with splinted attachments
4	Schincaglia et al. [21]	2016	No patient-related outcome
5	Scala et al. [22]	2012	Not involving 2-implant-retained overdentures
6	De Kok et al. [23]	2011	Compared directly with implant-supported fixed prosthesis
7	Akoglu et al. [24]	2011	Only ball attachments compared
8	Büttel et al. [25]	2012	Study type not RCT
9	Sun et al. [26]	2014	Not standardized attachments used
10	Ribeiro et al. [27]	2015	No patient-related outcome
11	Bryant et al. [28]	2014	Only ball attachments compared
12	Salman et al. [29]	2019	Only Locator attachments compared
13	Della Vecchia et al. [30]	2017	No patient-related outcome
14	Uçankale et al. [31]	2010	Compared directly with splinted attachments
15	Grandi et al. [32]	2012	Study type not RCT
16	Elsyad et al. [33]	2013	Compared directly with splinted attachments
17	Schuster et al. [34]	2020	Only Equator attachments compared
18	Giannakopoulos et al. [35]	2017	Only Locator attachments compared
19	Kappel et al. [36]	2016	Compared directly with splinted attachments
20	Geckili et al. [37]	2010	Only Locator attachments compared
21	Hasan et al. [38]	2016	Not involving 2-implant-retained overdentures
22	Pan et al. [39]	2010	Only ball attachments compared

**Table 3:** Summary of different characteristics and findings of the studies are included.

Authors	Year	No. of patients	No. of follow-up years	Types of attachments	Attachment types with better performance	Implant manufacturer	Implant types	Maxillary arch	Loading protocols	OHIP-14	Satisfaction	Comfort	Speech	Appearance	Chewing ability	Denture stability/ retention	Postinsertion maintenance
Bilhan et al. [40]	2011	Self-aligning abutments, n = 13 Ball abutments, n = 12	3 months for each attachment	Self- aligning vs ball	Self-aligning attachments are comparable to ball attachments in OHRQL and may be superior in cases of reduced space for attachment placement.	Osseospeed, Astra Tech	Standard 4.5 x 13 mm	Complete denture	Early (6 weeks after surgery)	All: no diff Below average space: ball > self	Nil	Nil	Nil	Nil	Nil	Nil	Nil

Krennmair et al. [41]	2012	Locator, n = 10 Crossover trial after 3 months	3 months for each attachment	Locator vs ball	No differences between ball or Locator attachment for any items of satisfaction evaluated and neither attachment had a significant patient preference Locator attachment required more postinsertion aftercare (activation of retention) than the ball anchors (nonsignificance)	Camlog, Screw-Line, Altatec	Standard	Complete denture	Delayed (3 months and 2 weeks)	Nil	No diff	No diff	No diff	No diff	No diff	No diff	No diff

Krennmair et al. [42]	2011	Ball, n = 13 Telescopic crown, n = 12	5 years	Ball vs telescopic	Frequency of technical complications was initially higher with ball attachments than with resilient telescopic crowns over a 5-year period	Camlog, Screw-Line, Altatec	Standard	Complete/ partial	Delayed (3 months)	Nil	No diff	n#il	No diff	No diff	No diff	No diff	Ball > telescopic; significantly more matrix repairs and activation

Cepa et al. [43]	2017	Ball, n = 12 Conus, n = 13	3 years	Ball vs conus	High dissatisfaction with the conus attachment resulted in numerous patients refusing to further participate in the study Except the deceased participants, all patients stayed with the ball attachment system, whereas only 7 of 11 patients stayed with the conus system Therefore, the investigated conus attachment system cannot be recommended	Ankylos, Dentsply, Germany	Standard	All three forms accepted	3 months	Nil	Higher with ball than conus	Nil	Nil	Nil	Nil	Nil	Nil

Jawad et al. [44]	2017	Standard, n = 22 Mini, n = 20	6 months	Mini-ball vs standard ball	No difference between both attachments	Mini-3M Standard-Astra Tech	Standard vs mini	Complete denture	2 months	Similar scores in both groups	No diff	No diff	No diff	Nil	No diff	No diff	Nil

## Data Availability

The data used to support the findings of this study are available from the corresponding author upon request.
